# Severe Spaghetti Wrist Injury With Complete Laceration of Median Nerve, Radial and Ulnar Arteries, and Multiple Flexor Tendons: A Case Report and Literature Review

**DOI:** 10.7759/cureus.105107

**Published:** 2026-03-12

**Authors:** Luis E Ocampo-Guzmán, Maria K Biviano-Andrade, Edgar E Esparza-Tapia

**Affiliations:** 1 General Surgery, Hospital Regional Universitario de Colima, Colima, MEX

**Keywords:** flexor tendon injury, median nerve injury, radial artery, spaghetti wrist, ulnar artery, zone v

## Abstract

Spaghetti wrist injuries are complex distal forearm lacerations involving flexor tendons, nerves, and vessels, associated with significant functional morbidity. We present the case of a 64-year-old right-handed man with a deep cut wound to his left forearm and clinical signs of distal ischemia, complete loss of digital flexion, and palmar anesthesia. An urgent examination was performed, identifying sections of the radial and ulnar arteries, median nerve, and injuries to the palmaris longus, flexor carpi ulnaris, flexor carpi radialis, superficial and deep flexor tendons of the fingers, and flexor pollicis longus tendons. Surgical repair followed by rehabilitation was performed. At six months, the patient demonstrated palmar sensory recovery and total active motion (TAM) of 73% compared with the contralateral hand, with residual limitation in digital flexion. This report highlights a severe spaghetti wrist injury with dual-artery transection causing distal ischemia and complete median nerve laceration, managed with single-stage primary tendon, vascular, and nerve repair, and reviews reconstructive options, repair sequencing, and rehabilitation strategies based on recent literature.

## Introduction

The term “spaghetti wrist” is used to describe extensive lacerations of the distal forearm and volar wrist that compromise multiple critical structures in the same plane: flexor tendons, major nerves, and vessels, with potential impairment of perfusion and overall hand function. Although its formal definition is not uniform, there is consensus that it is a highly complex clinical-surgical entity, where the timing of treatment, structural repair by planes, and protocolized rehabilitation determine the functional prognosis [1]. The objective is not merely wound closure, but functional restoration via tendon repair, revascularization in the presence of distal ischemia, and nerve repair, alongside rehabilitation to reduce stiffness and adhesions [1,2].

The severity of the damage is related to the number of structures injured and the presence of major neurovascular injury. In contemporary series, functional outcomes show variability, with frequent residual deficits, especially when there is complete nerve injury or associated ischemia, which reinforces the need for detailed reports of surgical technique and functional follow-up [3]. Furthermore, comparisons between studies are limited by the heterogeneity of assessment instruments (e.g., mobility, strength, sensitivity, and functional scales), particularly in zone V injuries, where the coexistence of multiple affected structures is the norm [4]. We present the case of a patient with a type 6 injury pattern [5] with extensive injury and double arterial repair, with sensory recovery and functional outcome, and contextualize it with relevant recent evidence.

## Case presentation

A 64-year-old right-handed man with no significant medical history presented to the emergency department with a deep cut to his left forearm (knife). Initial examination revealed absent distal capillary refill, with clinical signs of hypoperfusion, and severed arterial vessels visible along the length of the wound. The hand was hyperextended, with no active flexion of any of the fingers. There was also loss of sensation in the palmar portion, consistent with median nerve injury.

Immediate control of the bleeding was initiated by direct compression, and urgent transfer to the operating room was determined, given the suspicion of major vascular injury and neurological and tendon repercussions. The estimated ischemia time was one hour from the event to surgical reperfusion.

During the examination, complete sectioning of the radial artery and ulnar artery was identified, as well as complete injury to the median nerve. In addition, injuries to the following tendons were documented: palmaris longus (PL), flexor carpi ulnaris (FCU), flexor carpi radialis (FCR), flexor digitorum superficialis (FDS), flexor digitorum profundus (FDP), and flexor pollicis longus (FPL).

**Figure 1:**
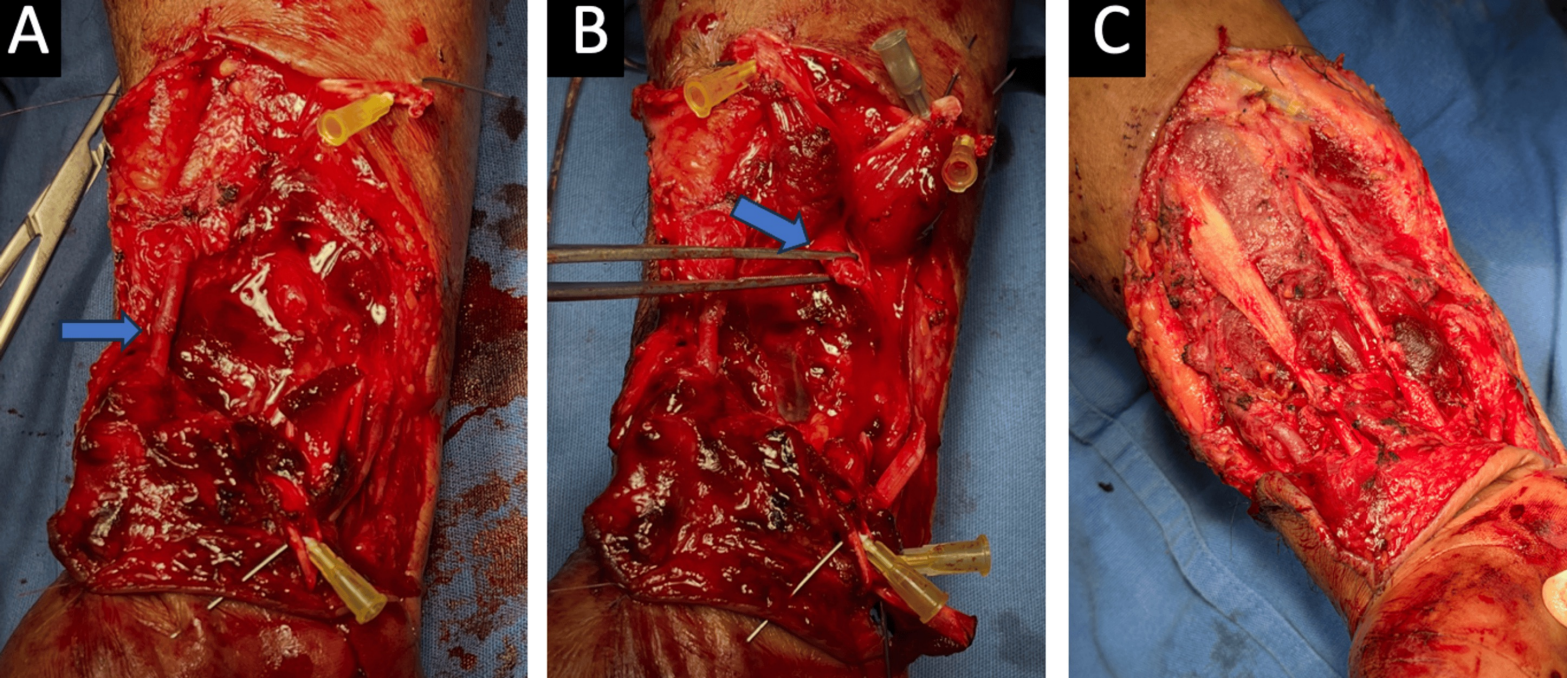
Spaghetti wrist (A) Radial artery anastomosis. (B) Median nerve injury. (C) Surgery completed with vascular anastomosis, neurorrhaphy, and tenorrhaphy.

Primary multistructural repair was performed during the same surgical procedure (Figures 1A-1C). Tenorrhaphies were performed using a modified Kessler technique with nonabsorbable monofilament suture (polypropylene, 5-0), selected according to tendon caliber and tissue quality. Subsequently, end-to-end anastomosis of the radial artery and ulnar artery was performed with 7-0 polypropylene. Neurological reconstruction consisted of epineural neurorrhaphy of the median nerve with 8-0 nylon.

Finally, a dorsal splint was applied, maintaining the wrist at 30° of flexion and the metacarpophalangeal joints at 60°, with the aim of protecting the repairs and optimizing the balance between tendon tension and reconstruction safety.

Given the multistructural tendon, nerve, and dual-artery repairs, the limb was immobilized for four weeks to protect the reconstruction. After this period, the patient began a rehabilitation program with passive mobilization and progression to active mobilization, under supervision, according to tolerance and clinical stability. At six months, palmar sensibility had recovered, and total active motion (TAM) reached 73% relative to the contralateral hand, with residual limitation in digital flexion. No major complications (infection, reoperation, tendon rupture, or vascular compromise) were observed during follow-up.

## Discussion

Spaghetti wrist injuries are considered reconstructive emergencies because they combine severe functional loss due to tendon and nerve damage with an additional risk of ischemia when there is major arterial injury. A recent review emphasizes that the lack of a universal definition does not reduce the clinical severity: these injuries require structured assessment of perfusion and function, complete surgical exploration and layer-by-layer repair, as well as early, protocolized rehabilitation as a determining factor in prognosis [1]. Reviews focusing on zone III-V injuries emphasize that the volar region of the distal forearm contains multiple tendons, nerves, and vessels, which explains the frequency of multistructural damage and the need for methodical planning to avoid intraoperative omissions [2].

In this case, absent distal capillary refill with visibly transected vessels raised concern for major arterial injury, prompting urgent surgical exploration. An ischemia time of one hour is considered clinically relevant: although the hand has collateral circulation, the simultaneous severing of the radial and ulnar arteries increases the risk of tissue hypoperfusion and secondary neurological deterioration. Contemporary clinical series on spaghetti wrist show that the number of injured structures is associated with a worse prognosis and that timely restoration of vascular continuity, together with stable tendon reconstruction, favors better functional recovery trajectories, especially within a multidisciplinary management with supervised therapy [3].

Complete injury to the median nerve usually causes much of the residual disability due to palmar sensory deficit and impaired fine motor skills. The return of sensitivity observed at six months in this patient suggests adequate coaptation and ongoing regeneration, although neurological recovery is expected to continue beyond this period. Recent literature highlights that technical decisions that minimize tension and optimize fascicular alignment are critical; contemporary meta-analyses have compared direct repair with assisted alternatives (e.g., connectors) in small gaps and discuss the potential impact on sensory outcomes, although the choice depends on the length of the defect, tissue quality, and the possibility of primary repair without tension [6]. In our case, primary epineural neurorrhaphy was feasible and clinically effective for initial sensory recovery.

In spaghetti wrist, the stability of tenorrhaphy must be balanced against the risk of stiffness and adhesions. In recent years, the highest level of evidence to guide rehabilitation has come from studies and trials on flexor tendon repair, comparing early active mobilization strategies with more passive protocols. A randomized clinical trial with long-term follow-up suggests that controlled regimens (including “place-and-hold” approaches versus active mobilization) influence functional outcomes and complications, highlighting the importance of individualization based on repair stability and risk of rupture [7]. In the case presented, four weeks of immobilization followed by progressive passive and active mobilization was chosen, a reasonable conservative strategy in the context of concomitant vascular and nerve repair; however, this approach may contribute to persistent decreased flexion, a common phenomenon in multistructural injuries due to adhesions and stiffness, even with adequate rehabilitation [1-3,7].

A recurring problem in the literature on zone V is the heterogeneity of outcome measures. Recent reviews point out that variation between studies (range of motion (ROM)/TAM, strength, sensitivity, functional scales) makes it difficult to compare results and set realistic expectations [4]. In our case, the patient has a mild limitation, which is favorable considering the initial severity and simultaneous injury to both main arteries and the median nerve. This finding is consistent with recent reports of severe cases, where functional recovery depends on complete repair, restoration of perfusion, and compliance with staged rehabilitation [8].

## Conclusions

Severe spaghetti wrist injuries with combined tendon, nerve, and vascular damage require prompt restoration of perfusion, meticulous single-stage multistructural repair, and structured rehabilitation. In our patient, palmar sensory recovery and TAM of 73% relative to the contralateral hand at six months were achieved despite residual limitation in digital flexion, supporting meaningful functional improvement after early reconstruction.
